# The role of the “beret” sign and other markers in ultrasound diagnostic of the acrania–exencephaly–anencephaly sequence stages

**DOI:** 10.1007/s00404-020-05650-y

**Published:** 2020-06-15

**Authors:** Piotr Szkodziak, Jarosław Krzyżanowski, Arkadiusz Krzyżanowski, Filip Szkodziak, Sławomir Woźniak, Piotr Czuczwar, Anna Kwaśniewska, Tomasz Paszkowski

**Affiliations:** 1 3rd Chair and Department of Gynecology, Medical University of Lublin, Lublin, Poland; 2 Chair and Department of Obstetrics and Pathology of Pregnancy, Medical University of Lublin, Lublin, Poland

**Keywords:** “Beret” sign, Neural tube defects, Acrania–exencephaly–anencephaly sequence, Prenatal diagnosis, Acrania, Exencephaly, Anencephaly, “Mickey mouse” sign, “Frog-eye” sign

## Abstract

**Introduction:**

Neural tube defects (NTDs) are a group of heterogeneous congenital anomalies of the central nervous system (CNS). Acrania is a non-NTD congenital disorder related to the CNS. It can transform into anencephaly through the acrania–exencephaly–anencephaly sequence (AEAS). In AEAS, the cerebral tissue is not protected and is gradually destroyed due to exposure to the harmful effect of amniotic fluid and mechanical injuries. These lead to exencephaly and then into anencephaly. In contrast to primary anencephaly (NTDs), this type of anencephaly authors suggests calling secondary anencephaly.

**Objective:**

Analysis of the known prenatal ultrasonography (US) signs associated with AEAS. Simultaneously, the authors propose a new sign in the differentiation of acrania from exencephaly and anencephaly, called the “beret” sign.

**Methods:**

It is a two-centre retrospective observational study. As part of the study, 4060 US scans were analyzed.

**Results:**

In 10 cases, the absence of calvarium was diagnosed, allowing recognition of either AEAS stages or primary anencephaly. In 5 cases, cerebral structures were enclosed by an inertial rippled thin membrane, with a smooth outer contour. Between the described membrane and the brain structures, a thin anechoic space corresponding to cerebrospinal fluid was observed. This sign was defined as the “beret” sign. In these cases, acrania was diagnosed. In three cases calvarium was missing. The brain structures had an irregular appearance, did not wave and remained motionless. The outer contour was unequal as if divided into lobes. Amniotic fluid was anechoic. Exencephaly was diagnosed in these cases. In two cases calvarium, brain structures, and meninges were missing. The “frog eyes” sign and slightly echogenic amniotic fluid were visible. In this case, anencephaly was diagnosed.

**Conclusions:**

The “beret” sign seems to be a promising tool in the diagnosis of acrania. Furthermore, echogenicity of amniotic fluid could be useful during differentiation between primary and secondary anencephaly.

## Introduction

Neural tube defects (NTDs) are a group of heterogeneous and complex congenital anomalies of the central nervous system (CNS) and, after heart defects, are the second most common fetal anomalies. They occur with a frequency of 1/100 to 1/1000 of pregnancies and have multifactorial origins. Nevertheless, a large percentage of NTDs is still undiagnosed. In Europe, the prevalence of NTDs stands at 2.3 per 1000 births [1].

Despite numerous studies, the aetiology of NTDs remains an issue that is not completely explained. Epidemiological studies indicate that their occurrence depends on genetic factors conditioned by multi genetic inheritance and environmental factors that could cause the expression of mutant genes [2–4].

The environmental factors that may increase the risk of NTDs include among others the influence of teratogenic chemicals, pregnant infections (Influenza virus, Cytomegalovirus, Rubella virus, Varicella-zoster virus, Toxoplasma Gondi), maternal diseases (diabetes, hypertension), overuse of anticonvulsant and antipyretic drugs, nutrients deficiency (acid folic acid, vitamin B12) and also genetic disorders of the fetus (Meckel–Gruber syndrome, Down's syndrome) [4–6].

The maternal age over 35 years is not an entirely understandable risk factor. The relationship between the maternal age and the incidence of NTDs could be illustrated by a U-shaped curve. The highest values of the curve are under 20 and over 35 years, and the lowest in the range of 20–29 years. However, the knowledge of this epidemiological relationship is still insufficient to prevent NTDs effectively. The introduction of advanced in vitro fertilization techniques among older women is associated with the need to carefully examine the impact of mother's age on the prevalence of NTDs [7]. Another similar risk factor of NTDs is kinship. Culture, religion, level of education and several other factors impact significantly on the prevalence and extent of relatedness in a given community [8]. Kinship is primarily associated with an increased likelihood of homozygosity and autosomal recessive disorders. Al-Gazala et al. observed an increased risk of NTDs in homozygous mothers with the C677T mutation in the methylenetetrahydrofolate reductase gene (MTHFR C677T) [9]. In some societies, relationships of relatives are still common. Thus, both the degree and the ways in which this factor influences the genesis of NTDs are still an interesting field for further research [1].

Studies of conception periods and their effects on NTDs indicate that in the Northern Hemisphere May and June have the highest NTS prevalence. The peaks can be caused by the intensity of solar radiation that induces oxidative stress in the body. It affects both the neural tube closure and the lateralization process [10]. Furthermore, overheating in the warmer months and feverish condition are probable risk factors for the development of NTDs [11].

Most of NTDs arise from disturbances in the neural tube closing process in early embryogenesis, up to 6th week of pregnancy. These defects are defined as dysraphia and are also called as an open NTDs [2, 3].

In the case of higher vertebrates, the neural tube is created by processes that form it, bend it and connect it to the neural plate. During its formation, the neural tube is sealed by a fusion in the dorsal median line. This closure prevents exposure to the external environment. In the case of an abnormal fusion, neuroepithelium is subjected to neuronal degeneration and deficit. The type and severity of open NTDs vary depending on the level of the body axis. Thus, disturbances occurring within the neural tube's cranial section lead to anencephaly or encephalocele, while those arising in the lower part of the neural tube cause the formation of spinal cord hernias [2, 6, 12].

Anencephaly is a lethal NTD based on the lack of the brain or most of its structures (in particular within the forebrain and cerebellum) [12, 13].

As mentioned previously, NTDs resulting from a primary neural tube disorder are exposed to the external environment, as opposed to necrotic NTDs, which are covered by the skin. Encephalocele and other lesions that are covered with the skin are examples of NTDs that arise after the neural tube closure. They are defined as closed NTDs [13, 14]. In these cases, nervous tissue in encephaloceles is connected to the brain through a narrow peduncle. About 70–80% of encephaloceles occur in the occipital region. Parietal and nasal encephaloceles are much less common [15].

Another type of congenital disorder, which is not NTD but is related to the CNS, is acrania. It is a rare lethal defect characterized by the absence of skull bones. The estimated incidence is approximately 1:1000 pregnancies [16].

In comparison to NTDs, which arise as a result of disturbances in the neural tube closing process, it is believed that acrania results from abnormal migration of mesenchymal tissue normally covering the cerebral hemispheres. An abnormality occurs at the beginning of the 4th week of pregnancy when the anterior neuropore closes. The lack of mesenchymal tissue displacement to the region of the brain's hemispheres results in the absence of skull bones. Excess of ectoderm remains the only cover of the brain in the form of a thin, amniotic membrane. Apart from the skull, also the muscles, scalp and dura mater are not developed. In the case of absence of induction from neurocranium, the cerebral tissue does not differentiate into two hemispheres. Finally, the cranial bones are partially or completely absent, with relative preservation of development of the cerebral hemispheres (although with abnormally developed brain tissue) [17, 18].

As fetal cranial ossification starts and accelerates after 9 weeks, prenatal US allows diagnosing acrania from the 11th week of pregnancy. According to the current recommendations, it is important to pay attention to the ossification of the frontal bone of the fetus in the axial and frontal planes [17–19].

Acrania could transform into anencephaly through the acrania–exencephaly–anencephaly sequence (AEAS) [20]. The theory of acrania evolution into anencephaly is broadly accepted by many authors. The first report about such possibility has appeared in the publication of Warren et al. in 1951 [21].

According to AEAS, the cerebral tissue that is not protected by the meninges, cranial bones and the skin is gradually destroyed due to exposure to the harmful effect of amniotic fluid (increased urea concentration in the amniotic fluid) and mechanical injuries (risk of friction with the uterine wall, placenta and fetal parts). This leads to exencephaly and then into anencephaly [20, 22, 23].

In this situation, the unprotected brain tissue is gradually destroyed and degenerated. It leads to complete or almost complete cerebral atrophy, starting from the 14th week of pregnancy. Destroyed brain tissue is suspended in the form of small fragments in the amniotic fluid, gradually increasing the echogenicity of the amniotic fluid [20, 24, 25]. In comparison to primary anencephaly (open NTDs disorder), this type of anencephaly authors suggests calling secondary anencephaly to AEAS. However, exencephaly is a lethal, congenital disorder of the fetal brain, which is characterized by a lack of calvarium and to various degrees of fetal brain tissue. It is considered as a direct precursor of secondary anencephaly [20, 25, 26].

AEAS allows understanding the much more frequent occurrences of anencephaly than acrania. The incidence of anencephaly in the European population is 3.52 per 10,000 births [27]. In the United States, this frequency is lower—9.40 per 100,000 births [28]. Some publications describe the conjugated incidence of anencephaly and acrania up to 1 in 1000 births [22].

In the cases of AEAS, the first trimester US there is a normal amount of brain tissue visible in the frontal plane of the fetus causing the appearance of the “Mickey Mouse sign” (“Mickey Mouse face”) due to two semi-circular structures hovering over the surface of the fetus, similar to the rounded ears of “Mickey Mouse”. This sign is typical for exencephaly. In the second trimester, a significant amount of brain tissue disappears, which manifests itself in the US as the “frog face” or “frog eyes” sign. This is caused by a lack of recognizable brain tissue above the level of fetal orbits. This term was also used by paediatricians in order to describe newborn infants who did not have forebrain and cerebrum, and the head was compared to the frog's head—the part of the skull and scalp were missing above the eyebrow line. This sign is typical of anencephaly [18, 29, 30]. Along with brain atrophy and transformation of the “Mickey Mouse” sign into the “frog-eye” sign (during the US), significantly increases the echogenicity of the amniotic fluid [20].

## Aim of the study

As it was mentioned in the introduction, apart from the assessment of ossification of the calvarium, the current literature describes two signs used in US diagnosis and differentiation of the fetal disorders resulting from the AEAS or the primary anencephaly. These are the “Mickey Mouse” sign and the signs of “frog face” or “frog eyes”. Moreover, ultrasound echogenicity assessment of amniotic fluid appears to be important in the diagnosis of AEAS [20, 29].

Nevertheless, many authors also emphasize that the recognition of the acrania–exencephaly–anencephaly sequence is difficult, especially during the early gestational age, and many cases remain undiagnosed at this stage of pregnancy [26, 31].

The aim of the study is to retrospectively analyze the known US signs associated with AEAS allowing the diagnosis and differentiation of individual stages of AEAS in the US. Simultaneously, the authors propose a new sign in the differentiation of acrania from exencephaly and anencephaly, called the “beret” sign [32].

## Material and methods

It is a two-centre retrospective observational study. As part of the study, 4060 US scans were analyzed. They were carried out in the first and second trimesters of pregnancy of patients hospitalized or consulted in the 3rd Department of Gynecology and Department of Obstetrics and Pathology of Pregnancy of the Medical University of Lublin in 2005–2019.

The patients were undergoing the US in two-dimensional (2D) and three-dimensional (3D) mode of the pregnant uterus with the assessment of anatomical structures of the fetus, with particular regard to the spine, spinal cord, brain, and skull. Bone ossification of the calvarium in the axial and frontal planes of the examined fetuses was also evaluated [17–19].

US scans were performed according to the Fetal Medicine Foundation (FMF) protocol [33].

2D scans using a transvaginal “convex” probe with a bandwidth of 5–9 MHz, field of view of 150.3 and 192-element convex array transducer were performed. In this mode, the transabdominal “convex” probe was also used with a bandwidth of 1–6 MHz (center of frequency 3.2 MHz), field of view of 60.61 and the number of elements equalled 128.

3D scans were performed with the use of volumetric transvaginal “convex” probe, with a bandwidth of 5–9 MHz (center of frequency: 6.5 MHz), field of view 150° and volume angle 90.0°. In addition, a volumetric transabdominal “convex” probe with a bandwidth of 4–8 MHz (center of frequency 4.4 MHz), with a field of view of 76° and volume angle 80.0° in 3D mode was used.

All of the images have been archived in electronic form or printed on thermal paper. 2D US scans were compared with 3D, as well as with the results of the visual autopsy of fetuses after a miscarriage/delivery.

Patients with suspected defects of the CNS within the fetal brain were qualified for the study.

The inclusion criteria were the diagnosis during the US an isolated defect in the fetal brain, being part of the AEAS or primary anencephaly.

The exclusion criteria included the diagnosis of encephalocele, closed NTD or fetal brain defects co-existing with other developmental disorders of the CNS, especially within the spine.

The research was approved by the local Ethical Committee of the Medical University of Lublin (KE-0254/305/2018), and each patient has a written informed consent to participate in the study.

## Results

The performed analysis revealed 15 cases of abnormal development of the CNS among fetus.

In 10 cases absence of calvarium in the sagittal and frontal plane allowing recognition of acrania, exencephaly or anencephaly (AEAS stages) or primary anencephaly was diagnosed. The symptoms were isolated only to the cranium. These patients (20–37 years, Caucasian, 12–16 weeks of pregnancy) were analyzed.

**Fig. 1:**
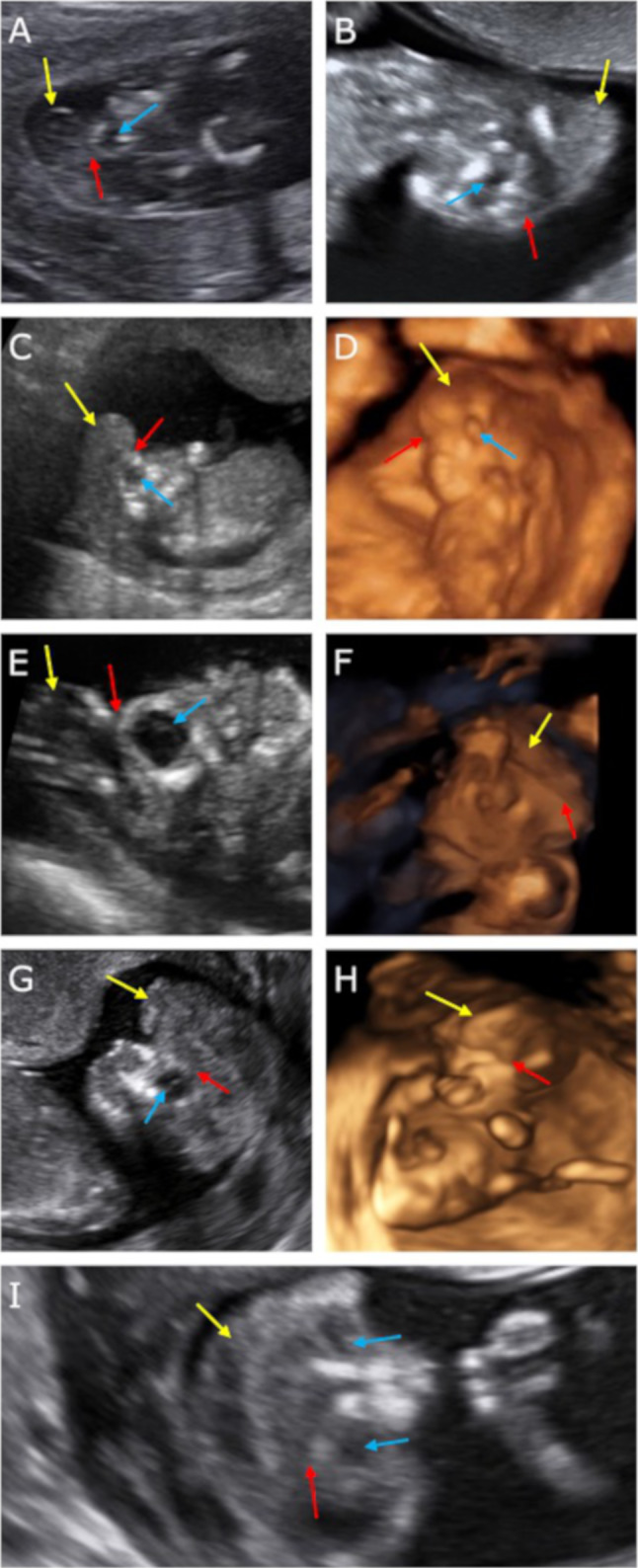
The US scans of pregnant women included in the study and described as cases 1–5. In these cases, acrania was diagnosed. Scans **a**, **c**, **d**, **e**, **f**—transabdominal probe and **b**, **g**, **h**, **i**—transvaginal probe. Scans **a**, **b**, **c**, **e**, **g**, **i**—2D and **d**, **f**, **h**—3D rendering. Scans **a**–**h**—sagittal cross-section and *i*—coronal cross-section. Yellow arrows—brain structures covered with rippling thin membrane similar to meninges—the “beret” sign. Blue arrows—orbits. Red arrows—bone edge on the border of calvarium defect

In five cases (the US made in the first trimester) both the sagittal and frontal cross-sectional views revealed cerebral structures enclosed thin inertially rippled membrane, with a smooth outer contour (2D/3D). Between the described membrane and the brain structures, a thin anechoic space corresponding to cerebrospinal fluid (2D) was observed. This structure was visible in the cranial vault spot (unlike encephalocele), protruding beyond the skull. In the frontal plane, the cerebral falx was not visible. This sign was defined as the “beret” sign. In these cases, acrania was diagnosed. The anechoic amniotic fluid was described (Fig. 1).

**Fig. 2:**
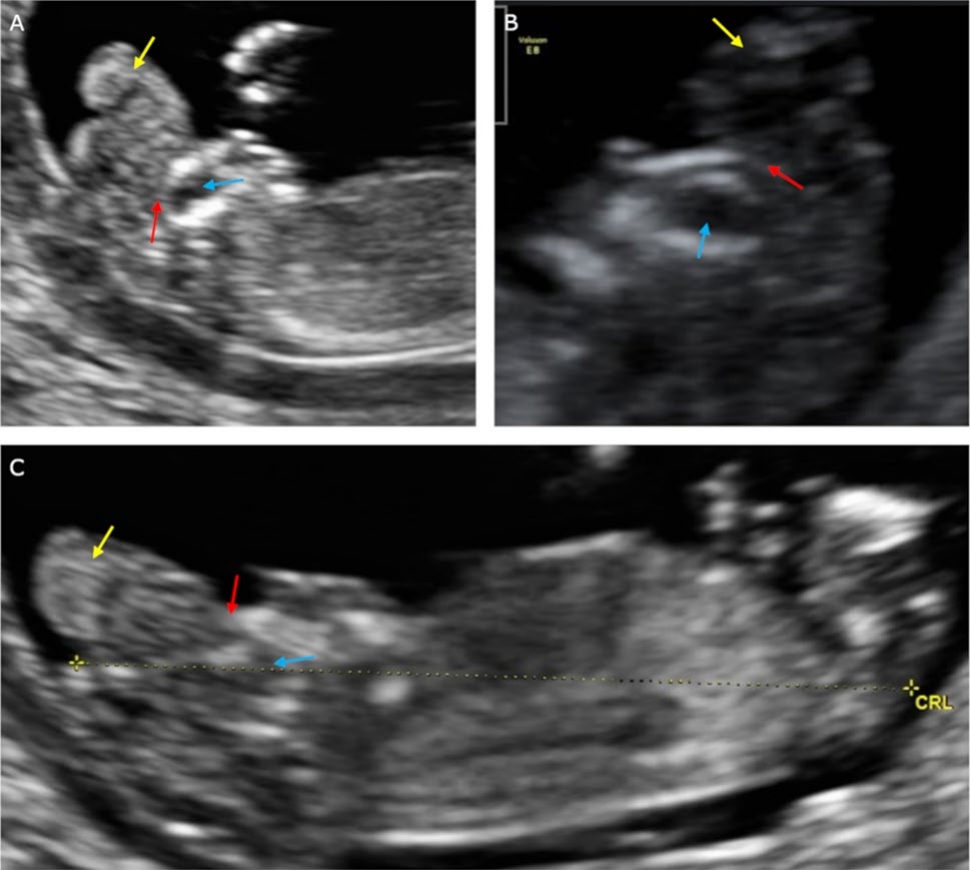
The US scans of pregnant women included in the study and described as cases 6–8. In these cases, exencephaly was diagnosed. Scans **a**, **c**—transvaginal probe and **b**—transabdominal probe. Scans **a**–**c** sagittal cross-section. Yellow arrows—brain structures divided into lobes with uneven external contour. Blue arrows—orbits. Red arrows—bone edge on the border of calvarium defect

In three cases (the US made in the first trimester) absence of calvarium was observed in sagittal cross-section. The brain structures were present but had an irregular appearance. The brain tissues did not wave and were motionless, while the outer contour was unequal as if divided into lobes. The described structures were also observed outside the skull (2D). The anechoic amniotic fluid was detected. Exencephaly was diagnosed in these cases (Fig. 2).

**Fig. 3:**
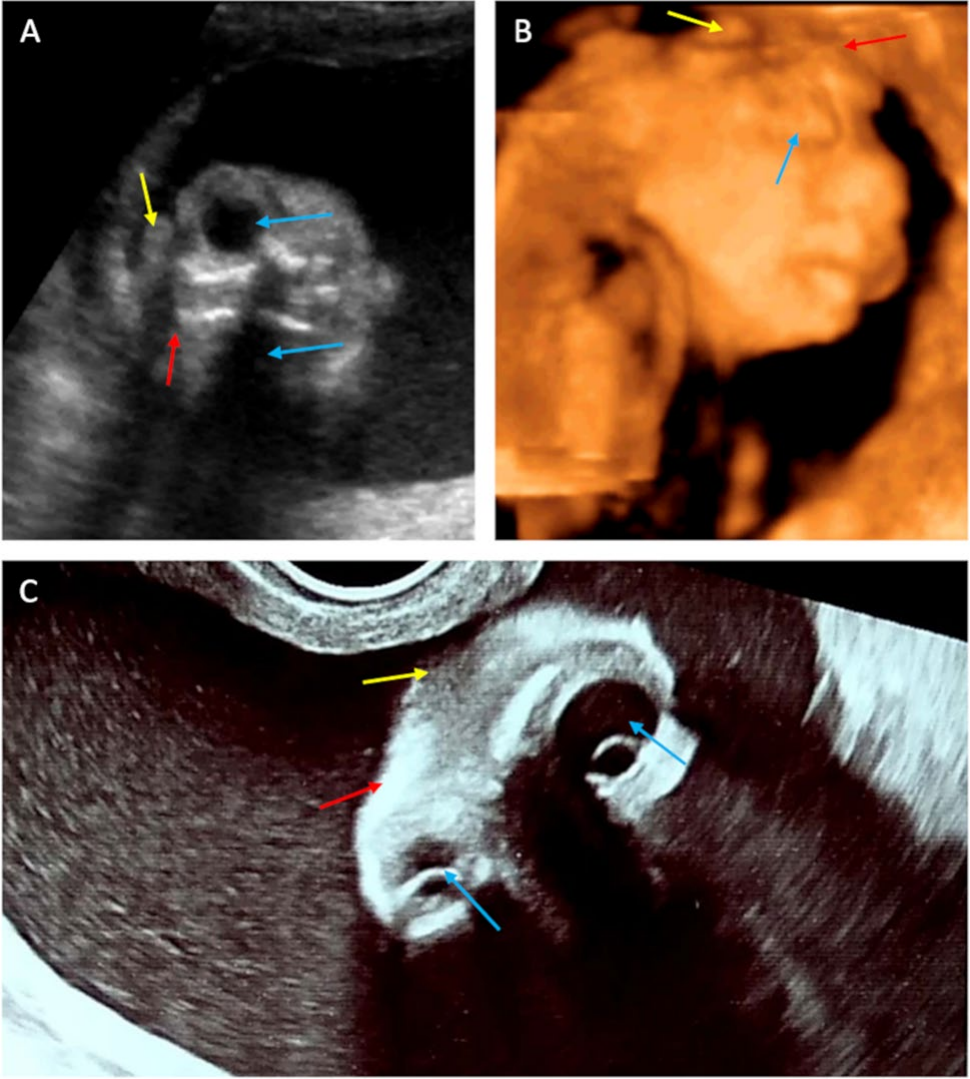
The US scans of pregnant women included in the study and described as case 9–10. In these cases, anencephaly was diagnosed. Scans **a**–**c** performed using the transabdominal probe in 2D (**a**, **c**) and 3D rendering (**b**). Scan **a**, **c**—coronal cross-section and scan, **b**—sagittal cross-section. Yellow arrows—degenerated brain structures. Blue arrows—orbits. Red arrows—bone edge on the border of calvarium defect

In two cases of the study group (the US made in the second trimester), there was an absence of calvarium, brain structures and meninges. Furthermore, the “frog eyes” sign (2D/3D), described in the literature, was detected [18, 29]. Moreover, slightly echogenic amniotic fluid was also found. In these cases, anencephaly was diagnosed (Fig. 3).

The above diagnoses were confirmed after the end of pregnancy.

Five patients were excluded from the study due to the coexistence of neurocranium defect and spina bifida (4 patients) or encephalocele (1 patient).

The characteristics of patients included in the study are described in the Table 1.

**Table 1:** Characteristics of patients included in the study

Case	Gestation/parity	Gestational age (weeks)	Cranial sonographic findings	US diagnosis	Amniotic fluid echogenicity	Autopsy
	Patient’s age (years)	Risk factors	Figure			
1	G3P1	12	“Beret” sign	Acrania	Anechoic	Acrania
	35	No	Figure 1a			
2	G4P2	13	“Beret” sign	Acrania	Anechoic	Acrania
	37	Caesarean scar pregnancy	Figure 1b			
3	G1P0	12	“Beret” sign	Acrania	Anechoic	Acrania
	25	CMV infection	Figure 1c, d			
4	G3P1	13	“Beret” sign	Acrania	Anechoic	Anencephaly (autopsy was performed at 32 weeks of pregnancy)
	30	Diabetes	Figure 1e, f			
5	G1P0	13	“Beret” sign	Acrania	Anechoic	Acrania
	21	No	Figure 1g–i			
6	G1P0	13	Disorganized brain tissue	Exencephaly	Anechoic	Exencephaly
	22	No	Figure 2a			
7	G3P1	13	Disorganized brain tissue	Exencephaly	Anechoic	Exencephaly
	29	No	Figure 2b			
8	G2P1	12	Disorganized brain tissue	Exencephaly	Anechoic	Exencephaly
	24	No	Figure 2c			
9	G1P0	16	Frog eyes	Anencephaly	Slightly echogenic	Anencephaly
	25	CMV infection	Figure 3a, b			
10	G1P0	25	Frog eyes	Anencephaly	Slightly echogenic	Anencephaly
	20	No	Figure 3c			

## Discussion

With the increasing popularity of ultrasound diagnostics in prenatal medicine, reports describing defects of the central nervous system of the fetuses began to appear. In 1972 Campbell et al. presented a case report of 17-week anencephalic infant based on analysis of the external contour of the head [34]. Analyzing the AEAS aetiology described in the introduction, the report is consistent with the generally accepted knowledge of the defect and relates to the lack of calvarium and diagnosis in the second trimester of pregnancy [20, 22].

The first ultrasound description of acranial fetus comes from 1986. Mannes e al. presented a case of an 18-week singleton pregnancy. In the US, calvarium was missing while spine and viscerocranium appeared normal. During the autopsy, cleft lip and missing frontal bone were described. In the discussion, it was noted that the presence of abundant but abnormal brain tissue, observed in the US, helped to distinguish acrania from more common anencephaly. The conclusions stated that exencephaly and encephalocele/cranioschisis should be included in the differential diagnosis, which is only possible after fetal autopsy [35].

In the opinion of the authors of this publication, figures attached to the Mannes et al. article allow for the diagnosis of exencephaly. In the US, the “Mickey Mouse” sign is visible, and the presented fetus after a miscarriage has no membranes covering the brain tissues. In addition, US was performed in second trimester, and according to the literature, exencephaly or anencephaly (primary or secondary) may be present during this period [20, 24, 25].

At the same time, we point out that the diagnosis of acrania in the US would be unlikely. The resolution of the ultrasound equipment used at that time probably did not allow the visualization of the “beret” sign [32, 35].

The first AEAS compliant description of exencephaly comes from 1994. Nishi and Nakano using vaginal probe described the “Mickey Mouse” sign at 11th week fetus. In coronal cross-section, cranial base and brain structures were visible without calvarium [30].

Amin et al. described a case of the singleton pregnancy consulted due to suspicion of CNS defect. The 30-week fetus examined in the US had a well-developed brain without cranial vaulting. Brain convolutions, interhemispheric fissure and sulci were clearly identified. The brain was covered with thin, rippling membranous structure. Under the influence of the probe's pressure, the fetal brain was easily compressed and seemed to float in the amniotic fluid above the cranial base. An increased amount of amniotic fluid has been described. The fetal brain showed normal vascular system evaluated in the colour doppler mode. No other fetal anomalies were visible. Cervical, thoracic and lumbar vertebrae appeared normal. Spinal canal and cord were of normal morphology. In fetal autopsy, calvarium was missing, viscerocranium appeared normal while the brain was covered with a thick membrane [36].

In the discussion, Amin et al. point out that ossification of the fetal cranium is not completed until the 10–11th week. Therefore, the first trimester US diagnosis must be careful, and thus fetal acrania can be diagnosed after 11th week of pregnancy. Between 11–14th weeks of pregnancy, most of the ossification points are found in lateral parts of frontal and parietal bones while the calvarium ossification is not visible in the mid-sagittal cross-section. Therefore, an incorrect diagnosis may occur if the US imagining covers only sagittal cross-section in the mid-sagittal line of the fetal cranium. This is the reason why, according to the quoted articles, it is important to evaluate the ossification of the frontal bone in axial and frontal cross-sections [37, 38].

**Fig. 4:**
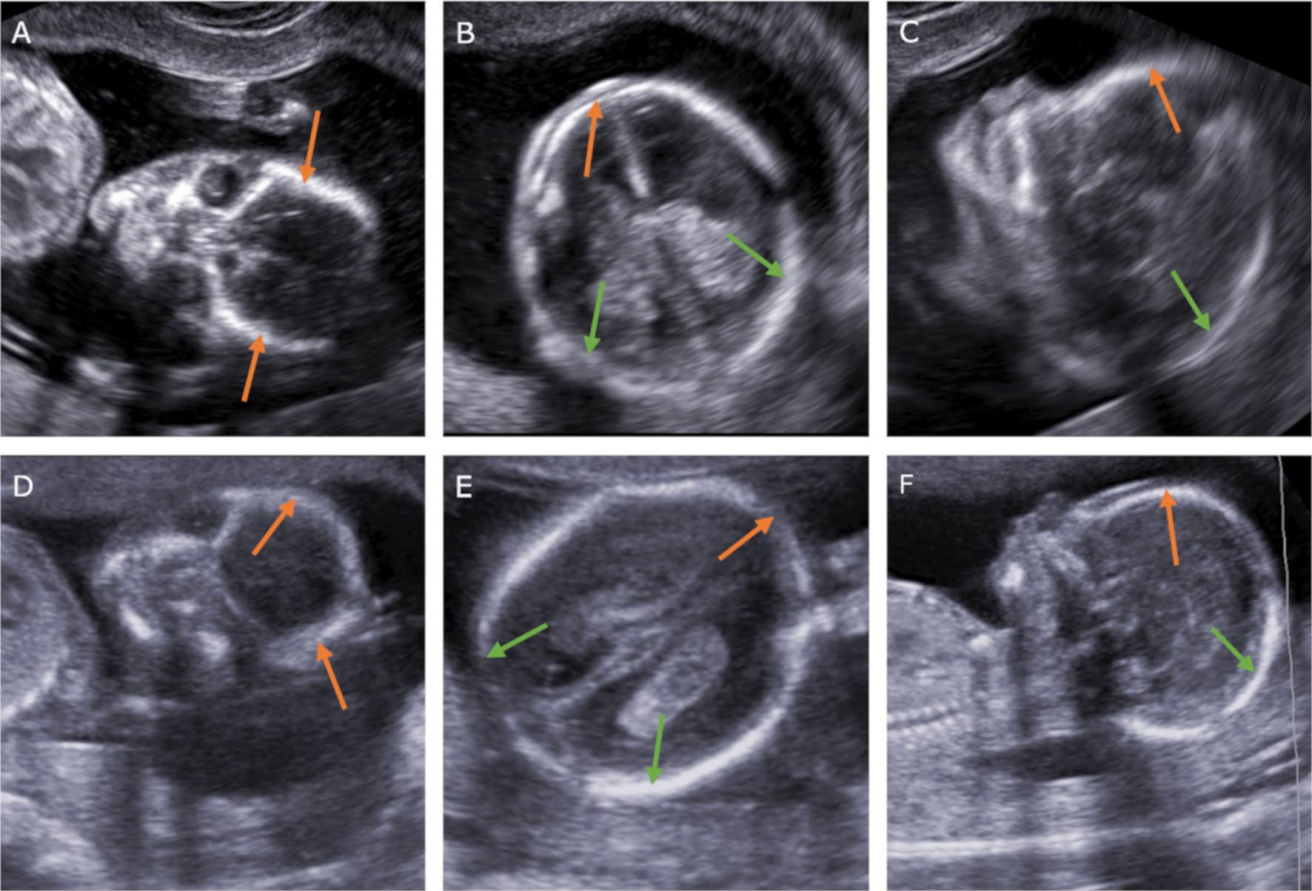
Ossification of the fetal calvarium. 12th week of pregnancy (transvaginal probe). Cross-sections: coronal (**a**), axial (**b**), sagittal (**c**) and in 16th week (transabdominal probe) in the analogue’s cross-sections (**d**–**f**). Orange arrows—frontal bones. Green arrows—parietal bones

Analyzing the above considerations, we came to a similar conclusion. The fetal US scans made in 12th week present normal views (Fig. 4). Sagittal (Fig. 4a), axial (Fig. 4b) and frontal (Fig. 4c) cross-sections present incomplete ossification of frontal and parietal bones forming the calvarium. Presented figures, however, do not give reason to diagnose acrania because of the lack of the “beret” sign, which would allow for differentiation of normal neurocranium from mentioned pathology. Images of the skull of a 16-week fetus in all presented planes show full ossification of the frontal and parietal bones (Fig. 4d–f), which is consistent with generally accepted knowledge [35–38].

In addition, all NTDs and stages of AEAS should be differentiated from severe osteogenesis imperfecta and congenital hypophosphatasia (type A, perinatal), which results in decreased ossification of the calvarium. In these cases, in which cranium is often deformed and difficult to differentiate from acrania, US may not be a reliable diagnostic tool. A family history of fractures helps differentiate [35, 36].

Doubts of the authors of this publication are caused by the diagnosis of acrania based on the case description presented by Amin et al. [36]. Membranous structures covering the fetal brain in 32nd week are the subject of objections. This is not in line with AEAS, where total or subtotal brain atrophy is observed from the 14th week of pregnancy [20, 24, 25]. In addition, careful analysis of the figures before and after delivery shows that brain structures are connected to the cranium by narrow peduncle and located in the occipital area, which is an example of closed NTD—encephalocele [18, 37].

Fong et al. provided a description of acrania compliant with AEAS. These authors point out that in the first trimester, the brains of acrania-affected fetuses may appear relatively normal or may exhibit varying degrees of distortion. However, an important feature in the US is the lack of calvarium. This feature allows diagnosis above 11th weeks of pregnancy. During 11–14th weeks, the lateral parts of the frontal and parietal bones undergo ossification, which cannot be seen in the mid-sagittal view. Therefore, diagnosis may be incorrect if ultrasound imagining covers only sections needed for NT measurement and nasal bone assessment. Using the “beret” sign, which is clearly visible in the midsagittal view, can limit the possibility of error in diagnosing acrania [37].

Engels et al. confirm the occurrence of AEAS. In the US they describe the “Mickey Mouse” sign that is visible in the frontal section. It reflects two divided hemispheres of the brain suspended freely in amniotic fluid. They link it with exencephaly. After progression to the stage of anencephaly the “frog eyes” sign appears in the US. It is usually observed in the second trimester [18].

Although the prenatal detection rate of anencephaly is almost 100%, the last study showed that in the Netherlands only about 69% of these defects are diagnosed before 18th week, and it is closely related to ultrasound training. Experienced ultrasonographers achieved a detection rate of only 86%, partly because some of the scans were performed before 11th week, which made it difficult to assess ossification of the calvarium. Authors of the quoted article did not give information about whether they were using other ultrasound markers of AEAS.

Weissman et al. were first (1997) to describe the acrania case compliant with AEAS. They analyzed and diagnosed five cases of acrania based on ultrasound images described as the coexistence of missing calvarium and a significant amount of disorganized brain structures covered only with a thin membrane. The authors concluded that thank to this symptom, acrania diagnostics can be carried out as early as the first trimester of pregnancy. Their description is consistent with the proposed “beret” sign. Weissman et al. did not link their findings to AEAS [19].

Liu et al. assessed the usefulness of US 3D in diagnosing acrania. They diagnosed 29 cases of fetal acrania. The range of gestational age in prenatal diagnostics in the US ranged from 11 to 21 weeks, of which 44% in the first trimester of pregnancy. Among them, 93.1% were isolated defects, and only one was associated with trisomy 18.

Compared with available reviews, they found that US 3D can detect fetal acrania as early as US 2D and can provide additional images obtained as a result of 3D reconstruction, which is not possible with US 2D. In conclusion, it was found that US 3D can contribute to the early detection of fetal acrania. Through 3D reconstruction, it enables innovative visualization of fetal defects, which helps make an early diagnosis [39]. The authors of this publication came to a similar conclusion by using US 3D in some cases. In the publication of Liu et al., there are only sample images of fetuses with acrania and exencephaly. Doubts are aroused by the lack of detailed descriptions or figures of the examined fetuses. As mentioned in the introduction of this publication, recognition of the acrania–exencephaly–anencephaly sequence is difficult, especially in the early gestational age. It seems reasonable to include descriptions, or US scans to verify if these were cases of fetal acrania, AEAS or other defects of the central nervous system [26, 31].

Santana et al. used US 3D to assess different phenotypes of AEAS. In the case of acrania, the phenotype has been described as a cystic, prolonged and irregular head shape, a description similar to the “beret” sign. In addition, the authors point out that the first symptom of AEAS may be abnormal echotexture of the amniotic fluid in the case of twin pregnancy, which is not consistent with the studies on single pregnancies. An interesting observation of Santana et al. is that exencephaly can be very well visualized at an early stage of embryonic development (in the 9th week of pregnancy) using US 3D. In this mode, it is possible to visualize the dimorphic features of the face associated with this pathology that are visible at a later stage of development such as low ears. The defect was confirmed during the fetal autopsy. In our opinion, this is an important application but requires further research. Additionally, the authors suggested that US 3D allows for a better understanding of brain malformations. It may be important in situations when the patient has doubts related to the diagnosis. As in the previously cited work, the methodology is unclear [20, 26].

Karasu et al. compared US 2D and 3D of the 11th-week fetuses with AEAS suspicion. In US 2D fetal calvarium had an irregular shape. In the upper part of the fetal head, ossified calvarium was missing, and brain structures were exposed to amniotic fluid. Both hemispheres and cerebral falx were visible. The 3D US image revealed an abnormal cranium. The fetal brain was present, but not covered by bone structures. The outer shape of the head was described as bi-lobed. Ultrasound in both modes (2D and 3D) indicates the “Mickey Mouse” sign allowing the recognition of exencephaly. The defect was confirmed during a fetal autopsy after miscarriage. In this case, the magnetic resonance of the fetus turned out to be non-diagnostic. In conclusion, Karasu et al. stated, that US 3D may help explain the extent and severity of acrania, both for doctors and patients [5]. The conclusion suggests that authors recognized acrania/exencephaly as the same stage of AEAS despite the descriptions corresponding with exencephaly.

As in the previously cited work, for diagnostic purposes, Hata et al. identified acrania/anencephaly as the same stage of AEAS. They analyzed 2 cases (fetuses in 13th and 17th week) using the US 3D and 4D (3D US in real-time) with HDlive rendering (with adjustable lighting and colour selection). The descriptions of both fetuses relate to exencephaly (the visible “Mickey Mouse” sign). In conclusion, Hata et al. stated that 3D/4D HDlive rendering mainly supports the understanding of the character of the defect. Furthermore, 3D/4D HDlive with skin-like colours gave the fetuses with acrania/exencephaly a natural and anatomically realistic appearance [40].

Cafici et al. described the US symptom indirectly associated with AEAS. They showed a link between the echogenic of amniotic fluid and fetal acrania in the US in the first trimester of pregnancy. In the conclusions, the authors confirmed a high rate of coexistence of fetal acrania and normal echogenic of amniotic fluid, suggesting that this finding could potentially be used as an AEAS marker. The finding also confirms the hypothesis of the evolution of fetal acrania, with the progressive destruction of the unprotected brain in the first trimester, to anencephaly in the second trimester. However, the criteria for diagnosing acrania include the absence of calvarium and poorly developed or disorganized brain structures, which corresponds more closely to the acrania/exencephaly phase in AEAS [20]. This observation was used as an additional fetal acrania marker in this study.

Currently, there is no direct method to distinguish between primary and secondary anencephaly. Based on the publication of Cafici et al. and our observations, it seems that there is an indirect method to perform this differential diagnosis [20]. Analysis of the echogenic of the amniotic fluid in the case of anencephaly can be helpful. In the case of anencephaly coexisting with the anechoic amniotic fluid, the diagnosis of primary anencephaly is reliable, whereas slightly echogenic amniotic fluid may indicate secondary anencephaly. This hypothesis requires further research.

In our study, the “beret” sign was visible in five fetuses, which allowed us to diagnose acrania. Amniotic fluid was anechoic. Analyzed US scans were made in the first trimester, which allowed for the diagnosis of the first stage of AEAS, thus complying with the data published in the literature [17–20].

In three fetuses examined in the first trimester, the brain was irregular, with an uneven external contour. The rippling, thin membranes covering the brain structures described above have not been visualized (the “beret” sign). Amniotic fluid was anechoic. In these cases, US was performed only in the sagittal plane, which precluded diagnosis of the “Mickey Mouse” sign. Further analysis of presented studies allowed for diagnosing exencephaly, which was confirmed during the fetal autopsy.

In two US scans performed in the second trimester, the lack of the “beret” sign and cerebral structures facilitated the diagnosis of anencephaly. Amniotic fluid was slightly echogenic. Lack of US performed in the first trimester of pregnancy made it impossible to differentiate between primary and secondary anencephaly clearly. However, slightly echogenic amniotic fluid may suggest secondary anencephaly.

The “beret” sign enables the diagnosis of the early stage of AEAS and the differentiation of acrania from exencephaly and anencephaly, which, as many authors emphasize, presents diagnostic difficulties in early pregnancy [26, 31].

## Conclusion

There are discrepancies in the available literature on the criteria for the diagnosis of AEAS in the US. Undoubtedly, attention is drawn to the fact that the accuracy of diagnoses increases with the use of modern ultrasound equipment, with a significantly higher imaging resolution and 3D/4D rendering abilities. The ability to determine the stages of development of the nervous system during embryogenesis consistent with the Carnegie Stages and the detection of these defects in ultrasound are essential for understanding pathogenesis and accurate diagnosis in clinical practice [41].

Acrania and remaining stages of AEAS are lethal NTDs. Differentiating it with treatable defects of the central nervous system will help to avoid wrong decisions on termination of pregnancy [15, 42].

The “beret” sign is visible in first trimester of pregnancy. During this time, amniotic fluid is anechoic. The “beret” sign is visible in the midsagittal and frontal section, which is a perfect complement to the bone ossification assessment of the cranial vault and seems to be a promising tool in the diagnosis of acrania.

Furthermore, US analysis of amniotic fluid could be useful during differentiation between primary and secondary anencephaly.

The coexistence of anencephaly with anechoic amniotic fluid suggests primary anencephaly, while slightly echogenic—secondary one.
